# Artificial Intelligence and Health Technology Assessment: Anticipating a New Level of Complexity

**DOI:** 10.2196/17707

**Published:** 2020-07-07

**Authors:** Hassane Alami, Pascale Lehoux, Yannick Auclair, Michèle de Guise, Marie-Pierre Gagnon, James Shaw, Denis Roy, Richard Fleet, Mohamed Ali Ag Ahmed, Jean-Paul Fortin

**Affiliations:** 1 Public Health Research Center Université de Montréal Montreal, QC Canada; 2 Department of Health Management, Evaluation and Policy École de santé publique de l’Université de Montréal Montreal, QC Canada; 3 Institut national d'excellence en santé et services sociaux Montréal, QC Canada; 4 Research Center on Healthcare and Services in Primary Care Université Laval Quebec, QC Canada; 5 Faculty of Nursing Science Université Laval Quebec, QC Canada; 6 Joint Centre for Bioethics University of Toronto Toronto, ON Canada; 7 Institute for Health System Solutions and Virtual Care Women's College Hospital Toronto, ON Canada; 8 Department of Family Medicine and Emergency Medicine Faculty of Medicine Université Laval Quebec, QC Canada; 9 Research Chair in Emergency Medicine Université Laval - CHAU Hôtel-Dieu de Lévis Lévis, QC Canada; 10 Research Chair on Chronic Diseases in Primary Care Université de Sherbrooke Chicoutimi, QC Canada; 11 Department of Social and Preventive Medicine Faculty of Medicine Université Laval Quebec, QC Canada

**Keywords:** artificial intelligence, health technology assessment, eHealth, health care, medical device, patient, health services

## Abstract

Artificial intelligence (AI) is seen as a strategic lever to improve access, quality, and efficiency of care and services and to build learning and value-based health systems. Many studies have examined the technical performance of AI within an experimental context. These studies provide limited insights into the issues that its use in a real-world context of care and services raises. To help decision makers address these issues in a systemic and holistic manner, this viewpoint paper relies on the health technology assessment core model to contrast the expectations of the health sector toward the use of AI with the risks that should be mitigated for its responsible deployment. The analysis adopts the perspective of payers (ie, health system organizations and agencies) because of their central role in regulating, financing, and reimbursing novel technologies. This paper suggests that AI-based systems should be seen as a health system transformation lever, rather than a discrete set of technological devices. Their use could bring significant changes and impacts at several levels: technological, clinical, human and cognitive (patient and clinician), professional and organizational, economic, legal, and ethical. The assessment of AI’s value proposition should thus go beyond technical performance and cost logic by performing a holistic analysis of its value in a real-world context of care and services. To guide AI development, generate knowledge, and draw lessons that can be translated into action, the right political, regulatory, organizational, clinical, and technological conditions for innovation should be created as a first step.

## Introduction

Artificial intelligence (AI) raises many expectations in all sectors of society. There is no universally agreed upon definition of what AI encompasses. Generically, it refers to a branch of informatics that develops systems that—through their ability to *learn*—imitate the characteristics associated with human intelligence: reasoning, learning, adaptation, self-correction, sensory comprehension, and interaction [1,2].

AI is seen as a strategic lever to improve access, quality, and efficiency of health care and services [3]. For example, by exploiting exhaustive data sets from complex systems, it could contribute to improving clinical decision making (eg, diagnosis, screening, and treatment), service organization (eg, flow optimization, triage, and resource allocation), and patient management and follow-up (eg, drug administration and compliance) [4].

However, research on the application of AI in health focuses primarily on technological performance in experimental contexts or on ethical issues. Although relevant, these studies do not fully address the broader systemic policy questions surrounding their use in a real-world context of care and services. In a recent meta-analysis, Lieu et al [5] concluded that despite a diagnostic performance equivalent to that of health care professionals, the diagnostic applications of AI have not been externally validated in a real-world context of care and services. Poor reporting is also prevalent in studies on AI, which limits the reliable interpretation of results. Thus, before being integrated into clinical routine, AI applications should overcome what is called the *AI chasm*, that is, the gap between reported performance in laboratory conditions and its performance and impacts in a real-world context of care and services [6]. AI raises issues of different types, but they are, in practice, closely interconnected: economic, professional, organizational, clinical, human, cognitive, legal, ethical, and technological. To date, few scholars have examined these issues in a systemic and holistic manner [7].

In this viewpoint paper, relying on the *health technology assessment (HTA) core model* [8], which is a methodological framework used to facilitate production and sharing of HTA information [9], we examine, based on our own experience as HTA academics and practitioners and in light of the emerging literature on the subject, issues raised by the use of AI. More specifically, we contrast the expectations specific to the health sector and the risks that should be mitigated for AI to be deployed responsibly. We limit our analysis to AI-based applications for clinical use (eg, diagnostic), some of which would be classified by the US Food and Drug Administration (FDA) as *software as a medical device*: “software intended to be used for one or more medical purposes that perform these purposes without being part of a hardware medical device” [10]. They are subject to formal regulatory approval [6,11].

In this paper, we offer critical observations and reflections that are informed by our various roles in HTA as health technology governance experts, researchers-evaluators, and/or decision makers. The analysis primarily adopts the perspective of payers (ie, health system organizations and agencies) because of their central role in regulating, funding, and reimbursing technologies [12].

On the basis of the *HTA core model,* we summarize key challenges posed by AI in a real-world context of care and services, which include (1) technological, (2) clinical, (3) human and cognitive (patient and clinician), (4) professional and organizational, (5) economic, and (6) legal and ethical dimensions (Textbox 1). We provide examples for each of these dimensions and underline how decision makers could approach them in a more systemic and holistic manner.

**Synthesis of some key challenges posed by artificial intelligence.**

**Technological**

- Laboratory performance versus a real-world context of care and services
- Data quality and representativeness of the general population or other contexts
- Black box: how and why the decision is made?
- Is artificial intelligence (AI) reliable and free of biases or technical failures?
- How AI would react in situations where input data deviate from initial data?
- Cybersecurity: data and model (algorithm)
- Interoperability: fragmented systems and unstructured data

**Clinical**

- Reproduction of tropism of practice models
- Actual clinical added value in a real-world context of care and services: difficult to distinguish the effect of the AI's decision from the rest of the preventive and/or therapeutic strategy
- The level of accuracy of AI in diagnosis and recommendations (reference standard) in a real-world context of care and services

**Human and cognitive (patients)**

- Evolution of the nature and quality of the clinician-patient relationship
- Loss of human contact: isolation of some people
- Unrealistic expectations in some patients regarding clinical outcomes
- Black box: could be perceived as a restriction on the patient’s right to make a free and informed decision
- AI could be beneficial for one part of the population and not be for others: what is the good target population?

**Human and cognitive (clinicians)**

- How to integrate AI into the electronic health record (EHR) and clinical routine with minimal effort and disruption for clinicians?
- Nonintuitive technologies: weigh-down workflows and burden for clinicians and cognitive overload
- Disruption of interpersonal communication styles (eg, clinician-clinician and clinician-patient)
- AI as clinical mind: challenge of clinician’s decision-making autonomy
- Absolute confidence in AI: technical dependence

**Professional and organizational**

- How will it fit into the patient care and services trajectory?
- How will it be integrated into the clinical-administrative processes and workflows of organizations and health system?
- What changes will result in terms of service organization (eg, waiting time, primary care and specialized services relationships)?
- How will it impact on professional jurisdictions (eg, reserved activities, responsibility, training, new skills, and expertise)?

**Economic**

- Investments required: continuous performance tests, software and data quality tests, infrastructure and equipment upgrades, human expertise, and training
- Clinical tropism and reimbursement/billing biases: costs for patients, clinicians, organizations, and health system
- Need of new financing mechanisms, appropriate remuneration and/or reimbursement models, and insurance models

**Legal and ethical**

- When is AI considered as a decision-making support tool? When is it considered as a decision-making tool?
- What are the limits of technology and their potential legal implications?
- If the AI makes a mistake (eg, black box), who will be held responsible? If the patient is harmed, who will pay for the repairs?
- What would be the consequence if the clinician does not comply with the recommendations of an AI and this leads to an error?
- AI needs access to data from different sources: consent is becoming more complex, as patients will be asked to authorize the use of diversified amounts of data
- Protection and confidentiality: origin of the data, how consent was obtained, and authorization to use and/or reuse the data
- Who owns the data? Who is responsible for it? Who can use (or reuse) it and under what conditions?

### Technological Dimension

#### Generalizability and Reproducibility

Studies that focus on technological issues indicate that AI should provide the same level of performance in a real-world context of care and services as that obtained in laboratory conditions. However, this requirement is difficult to achieve [13-16]. The majority of AI applications reported in the literature are not exploitable in clinical practice [17]. AI is often trained with so-called *clean* (exclusion of poor-quality images) and complete data sets (elimination of imperfect data) [18]. It may not be operational in other contexts where data are incomplete or of poor quality (electronic health record [EHR] with missing data and/or erroneously entered data) [19-21]. This applies to some categories of the patient population (eg, low economic status and psychosocial problems) who receive care and services in a fragmented way in several organizations (institutional wandering) [21-24]. In addition, AI is usually trained on data specific to certain sites (hospital) and patients who are not necessarily representative of the general population. This includes decontextualized data (lack of psychosocial and organizational indicators) and data about disproportionately sick individuals (data enriched by metastases cases), men, and those from a European origin (ethnodiversity) [23,25-28].

Health organizations and systems produce and manage data in different ways. Variations may exist in clinical protocols (eg, diagnosis, procedures, and vital parameters) and devices (eg, different types of scanners, EHRs, and laboratory devices) on which AI applications are trained and those on which they are expected to operate [29,30]. These variations could affect the AI performance in a real-world context of care and services [31]. For example, an AI application trained on data from 2 hospitals in the United States performed poorly in a third hospital [13,32]. In its decision, the AI application had as predictors the image characteristics (magnetic resonance imaging machines specifications), imperceptible to humans, specific to the technological systems of the hospitals where it was trained. The AI solution had adapted to *noise* rather than to the signal of clinical interest [33]. When used in the third hospital, it was *deprived* of these expected predictors (*noise*), which affected its anticipated performance [32]. In the same vein, the use of data from the *Framingham Heart Study* to predict the risk of cardiovascular events produced biased results, which both overestimated and underestimated risk when AI was used in non-white populations [34,35]. The ability of AI to operate without bias or confounding factors on different devices and protocols remains a major challenge [36,37]. Thus, the fact that an algorithm was trained on large data sets does not mean that its results are generalizable.

#### Interpretability and Transparency

The interpretability and transparency of AI are important issues. The *black box* logic makes some AI applications vulnerable and at risk to false discoveries via spurious associations: how is the decision made and on what basis (justification and process description) [24,38,39]. This issue is central because these technologies will be diffused on a large scale. The error of a defective AI could have a greater impact (several patients) than a clinician's error on a single patient [20,31,40].

Interpretability and transparency are also necessary to identify the origin of errors, biases, or failures that should be prevented and/or avoided in the future [3,21,41]. For example, an AI application could lead to many undesirable impacts related to: (1) poor-quality training data, which could lead to erroneous or biased knowledge (*garbage in, garbage out*), whereas technology may further amplify how poor data produce poor results (noisy data and missing values); (2) the presence of a technical flaw in the algorithm (code), which could lead to erroneous inferences, even if good-quality data are used; (3) decision-making criteria that may not be universally acceptable; and (4) the emergence of new situations for which AI could not adapt, even with good-quality data and code [21,30,42-45]. For example, the emergence of new treatments or practices may require changes in clinical protocols; however, at present, AI applications are not developed to manage temporal data naturally in a real-world context of care and services. However, diseases and treatments evolve in a nonlinear manner [18,45]. The question thus remains regarding how AI would react, with observable indicators, in situations where input data deviate from initial data (EHRs and real-time monitoring devices), in the medium and long term [45,46].

The risk of cyberattacks is also a major concern. The data could be modified and/or fed by other false or biased data in a way that is difficult to detect [1]. For example, a slight intentional modification of laboratory results in a patient's EHR resulted in significant changes in the estimates of a *well-trained* AI of the same patient's risk of mortality [24]. For AI, the issue is two-fold because it is necessary to ensure the security of the data and that of the model (the algorithm). Interoperability is also a significant issue. The integration of AI in fragmented and noninteroperable information technology systems and organizations could create more problems than it will solve; to deliver its full potential, AI needs integrated and interoperable systems with fluent and optimal data circulation and exchange [17].

Finally, addressing interpretably and transparency in AI could be compromised by intellectual property issues, competitive strategy, and financial advantages that make companies reluctant to disclose their source codes [3].

### Clinical Dimension

AI can entrench and disseminate practice models specific to particular contexts (organizations or health systems) and not necessarily accepted or used in others (tropism) [38]. For example, clinicians in some countries stopped using *IBM Watson for Oncology* because it reflected US specificity in cancer treatment [1,47].

To use AI in their decision making, clinicians should understand how it makes decisions in the first place [38,45,48]. They need the evidence to support a given conclusion to be able to carry out the necessary verifications or even corrections [14]: Why this decision (what information or image—or part of the image—tipped the final decision of the AI)? Why not another option (or choice)? When may I consider that the decision is correct? When should I accept this decision? How can I correct the error when it occurs?

AI should provide clinically added value for the patient. In a real-world context of care and services, much information, decisions, and diagnoses could intersect (eg, symptom assessment, laboratory tests, and radiology). At present, it is difficult to distinguish the effect of an AI-based decision from the overall preventive and/or therapeutic strategy of patient care [49,50].

Another clinical issue is determining the level of accuracy of AI for diagnosis and recommendations. In practice, decisions physicians make could diverge or even contradict each other in many situations. The *gold standard* is not always easy to define in a process that involves complex judgments [38,51,52]. In this case, should the standard reflect that of the lead clinician (or clinicians) in the organization? Or the one accepted by the majority of clinicians? Or the one reported in similar contexts? Some authors believe that for technologies that aim to provide pragmatic solutions under suboptimal conditions, AI performance should correspond to clinically acceptable practice in a given context and not necessarily to recommended practices [32]. This last point is likely to be problematic, particularly in a context where health systems are trying to overcome the challenge of practice variations to be able to provide equitable and quality services for all citizens.

### Human and Cognitive Dimensions

AI could affect the nature and quality of the clinician-patient relationship and their expectations for care and follow-up [53,54]. The loss of human contact could lead to increased isolation of some people (replacement of health care providers) [1]. Some patients may feel able to control and manage their disease, with passive surveillance and/or less contact with the clinician, whereas others may feel overwhelmed by additional responsibilities [55]. AI may also create unrealistic expectations in some patients regarding clinical outcomes, which could have a negative impact on their care and service experience [56]. In addition, some AI-based decisions could be perceived as a restriction on the patient's right to make a free and informed decision [1,53]. Cultural and social aspects could play an important role in how patients will respond to AI and therefore how effective it can prove in practice [57]. Hence, it is important to know on which basis one may define the target population that can benefit from it [58]. In this regard, the question of social acceptability (acceptable risk and public confidence) also needs to be considered, which goes beyond the simple question of the effectiveness and usability of AI [59].

For clinicians, the challenge is to integrate AI into the EHR and clinical routine with minimal effort while respecting their decision-making autonomy [24]. Nonintuitive technologies could encumber workflows and become a burden for clinicians without improving service delivery [30,60]. Otherwise, the ability of AI to combine data from the scientific literature with learning from practice data could generate a repository of clinical practices (*clinical mind*), which could give AI an *unwanted* power or authority [35]. In some situations, AI may reduce the clinician's ability to take into account patient values and preferences. In contrast, some clinicians may develop absolute confidence and become dependent on AI, thus relinquishing their responsibility to verify or double-check its decisions [1].

In short, if clinicians feel overloaded and workflows become more complex, AI may be rejected because of self-perceived inefficacy and performance, alert fatigue, cognitive overload, and disruption of interpersonal communication routines [54,61-63].

### Professional and Organizational Dimensions

Global appreciation of the added value of AI should take into account the nature and magnitude of the professional and organizational changes required for its use [6]. For example, the FDA has approved an AI application used for diabetic retinopathy screening, which may be used in primary care clinics [11]. As in some countries, the screening procedure is performed by an ophthalmologist (specialist), some questions arise: How will this technology fit into patient care and services trajectory? How will it be integrated into the clinical-administrative processes of organizations and the health system? If used at the primary care level, will general practitioners, nurses, or optometrists be allowed to supervise the AI? If so, under what conditions? What will be the impact on professional jurisdictions (regulated activities, remuneration, and training)? What changes will result in terms of service organization and clinical-administrative workflows (waiting time at primary care level, primary care, and specialized services relationships)?

Thus, AI could lead to a redistribution of work between different professional scopes of practice and highlight the need for other clinical, administrative, and technical skills and expertise. This will require clarifying new rules and processes (clinical and administrative), negotiating and reframing professional jurisdictions, responsibilities, and privileges associated with them and reassessing the number of positions needed and the new skills required to work (with) and/or perform other tasks that accompany its use. This will have to take into consideration how new roles in terms of skills in informatics and data science and the ability to liaise may be introduced within clinical teams [64].

Finally, today, most AI applications are developed to perform a single task or a set of very specific tasks (eg, diagnosing only diabetic retinopathy and macular edema) [65]. They are unusable for other diagnoses for which they are not trained (eg, nondiabetic retinopathy lesions and eye melanoma) and are unable, at least for the moment, to replace a complete clinical examination [66]. Payers will thus be tasked to determine whether AI provides sufficient added value in relation to the nature and magnitude of the clinical, cognitive, professional, and organizational changes it could generate.

### Economic Dimension

To adapt an AI to a local environment, considerable investments and expenditures may be necessary. The evolution of AI in a real-world context of care and services, by integrating large amounts of data of various types and sources, requires additional resources to ensure its proper functioning and stability: continuous performance tests, software and data quality tests, infrastructure and equipment upgrades, human expertise, and training [3,67]. However, many health organizations do not have a secure and scalable technological and data infrastructure as well as adequate human resources to ensure proper collection of the data necessary for the training and adaptation of AI to their local population and clinical environment [17]. The literature on AI’s promises as well as innovation policies that support its development downplays the capital-intensive requirements that are required to properly deploy AI, compared with the day-to-day work of managers in organizations.

In health systems where activity-based financing is the basis for funding health organizations, some clinicians tend to enter the *highest paying* codes for each clinical activity (ie, the most complex case of an intervention) to increase performance and maximize revenue. An AI application trained on data from these organizations (EHR with invoicing or reimbursement data) could amplify biases inherent in such practices that do not necessarily reflect the actual clinical condition [23,44,68]. The replication and entrenchment at a large scale of these biases could result in significant costs for patients, clinicians, organizations, and the health systems [35].

Similarly, some AI applications may be *too cautious*, resulting in an increase in requests for unnecessary testing and treatment, leading to overdiagnosis or overprescription [69]. Their recommendations, which are not necessarily associated with improved patient outcomes, could lead to increased costs and expenses for patients and the health system.

### Legal and Ethical Dimensions

Many AI technologies are still considered today as *decision-making support tools* for clinicians. It could then be argued that the legal responsibility for the decision still rests with the clinician. However, with the growing performance of AI, clinicians may be increasingly influenced and may more easily accept AI decisions, even when there is clinical ambiguity. Determining the clinician's degree of responsibility becomes more complex [30]. The challenge here is to distinguish between several situations: When is it considered a decision-making support tool? When is it considered a decision-making tool? This distinction is key in defining who is legally responsible in the event of an error or a malfunction (professional misconduct) [30,51,70].

For example, if the clinical decision is based on an erroneous clinical recommendation from the AI (delayed or erroneous treatment), who will be held responsible? Is it the technology developer, technology provider, clinician, organization, or do they all share responsibility (and how)? In some jurisdictions, to confirm professional misconduct, it is necessary to prove that the *standard of care* was not followed. This standard is blurred when AI comes into play [2]. In addition, the likely consequence if the clinician does not comply with the recommendations of an AI and if this leads to an error must be anticipated [2]. It could be argued that the responsibility should rest with the human controller of AI, but such a responsibility becomes difficult to clarify when autonomous technologies are used [57]. In this regard, standards may shift over time: “What happens if medical practice reaches a point where AI becomes part of the standard of care?” Medical insurers and regulators will have to be able to distinguish errors inherent in the tool from those resulting from misuse by the clinician, the organization, or even the patient, an issue exacerbated by the *black box* of AI [51,71].

To generate a complete picture of the patient, AI will need access to data from different organizations (hospitals and insurers) [45]. The risk of disclosing sensitive information about patients or certain populations is real [45]. For example, some AI applications can reidentify an individual from only three different data sources [25,38,72]. In the same vein, the issue of consent is becoming more complex, as patients will be asked to authorize the use of increasingly large and diversified amounts of data about them: medical records, audio, videos, and socioeconomic data [58]. Problems could arise if the patient only consents to sharing parts of his or her data. Usually, confidentiality means that the clinician can withhold certain information—at the patient's request (or not)—and avoid entering it into the EHR. Incomplete data make AI less efficient and does not allow patients to benefit from the best possible services. AI may not be fully operational in a real-world context of care and services if specific restrictions on data access and use are applied [38].

Protection and confidentiality requirements imply the obligation to know several things: the origin of the data, how consent was obtained, and authorization to use and/or reuse the data for training and in a real-world context of care and services. As the data may come from different sources and contexts, different conditions and precautions will need to be considered [73]. Regulators will need to determine who owns the data and, in the context of public-private partnerships, who is responsible for its collection, use, transmission to third parties, and under what conditions [17]. As the answers will vary according to the nature of the data, the jurisdictions, and the purpose of use, the task at hand is sizable [73]. Finally, payers will have to recognize that the ethical implications of AI affect, directly or indirectly, all the other dimensions discussed earlier.

### Conclusions

The purpose of this viewpoint paper is to provide a structured roadmap of the issues surrounding the integration of AI into health care organizations and systems. To the best of our knowledge, this is one of the few papers that offers a multidimensional and holistic analysis on the subject [7]. It contributes to current knowledge by providing a necessary basis for reflections, exchanges, and knowledge sharing among the various stakeholders concerned with AI in health care.

In light of the issues we identified, it becomes clear that regulatory and decision-making organizations as well as HTA agencies are facing unprecedented complexity: evaluating and approving so-called disruptive technologies, especially AI, requires taking several issues into consideration altogether. Many studies have reported significant technical performance of AI technologies, but very few have adopted a holistic standpoint that can situate their impacts and associated changes and transformations in health systems. Technical studies are rarely adapted to the complexity surrounding AI applications, as they overlook the context-dependent changes or adjustments the implementation and use of technology requires (variations, clinical and organizational interactions, and interdependencies) [74]. According to *the frame problem* [62,75], which highlights the difficulty for AI, beyond the specific tasks it masters, to update its set of axioms to capture the context in which it is implemented and used (eg, patient preferences, environment and social support, clinical history, personality/cultural characteristics and values that influence clinical outcomes, and empathy in medicine), the complexity inherent in the use of AI applications in the real-world context of care and services may seem difficult to overcome [62].

For informed decision making, there is a real need for evaluations that address AI as a lever of health system transformation. Given the magnitude of the implications it could have at all levels, the evaluation of AI’s value proposition should go beyond its technical performance and cost logic to incorporate its global value based on a holistic analysis in a real-world context of care and services. In this vein, technology brings value when its use in a real-world context of care and services contributes to the aims of the health system and aligns with the values of society. Global value appreciation could be based on the *quintuple aim*: (1) better quality and experience of care and services for patients; (2) a better state of health and well-being for the entire population; (3) reducing costs for responsible and sustainable resource management; (4) a better quality of work and satisfaction of health care providers; and (5) equity and inclusion to avoid exacerbating health disparities in the population [76]. From this perspective, further research on the evaluation of AI should no longer be limited to a technological approach, which only demonstrates quality from an engineering point of view and costs—motivated mainly by a logic of short-term savings—but should broaden its horizons to include the dimensions this paper underscored [77,78].

Real-world evaluations could be a major asset in informing AI decision making. In the context of uncertainty, iterative and reflective evaluation approaches should be developed to encourage dialog and collaboration among all relevant stakeholders (eg, payers, health care providers, technology providers, regulators, citizens/patients, academic researchers, and evaluation agencies) [63,78,79]. In addition, an early dialog between these stakeholders is needed to identify the evidence required to inform decision making [63,78]. This approach would also help AI providers to better understand the expectations of the health system [78]. This change implies that HTA should play an active role as a mediator and facilitator of transparent dialog between different stakeholders who are implicated throughout the technology’s life cycle [78,80].

Decision making for innovative technologies is inherently complex, in particular because of visions, perceptions, and objectives that may differ between the stakeholders involved: *risk sharing* is essential to strive to find a balance between uncertainty and added value [81]. In this regard, “major radical innovations never bring new technologies into the world in a fully developed form” but “appear in a crude and embryonic state with only a few specific uses” [81]. It is their use in a real-world context of care and services, through a process of *learning by doing* (improving users’ skills) and *learning by using* (improving users’ knowledge), which makes it possible to appreciate their global value [81]. With the complexity associated with AI, value appreciation becomes even more complex, challenging the traditional methodological foundations that are the basis for decision making about innovative technologies [82]. This also presents a unique opportunity for HTA to evolve and adapt (evaluative framework and contextualized data), particularly in view of the importance of contexts in the appreciation of the value of innovative technologies [83,84]. It is necessary for HTA scholars and practitioners to explore and exploit other avenues, complementary to traditional methods, to collect data and information that can better inform AI-related decisions [85].

Finally, this new context implies mechanisms for continuous collective learning and sharing of lessons. To do so, there is a need for learning and flexible health organizations and systems that are able to adjust and operate under uncertainty. In this regard, creating the political, regulatory, organizational, clinical, and technological conditions necessary for proper innovation is the first step. This requires building trust to ensure stakeholder engagement to guide AI developments, rapidly generate knowledge in a real-world context of care and services, and draw lessons to translate them into action.

## Abbreviations

AI: artificial intelligence
CIHR: Canadian Institutes of Health Research
EHR: electronic health record
FDA: Food and Drug Administration
HTA: health technology assessment
